# *Xanthomonas oryzae* Pv. *oryzicola* Response Regulator VemR Is Co-opted by the Sensor Kinase CheA for Phosphorylation of Multiple Pathogenicity-Related Targets

**DOI:** 10.3389/fmicb.2022.928551

**Published:** 2022-06-09

**Authors:** Lulu Cai, Wenxiu Ma, Lifang Zou, Xiameng Xu, Zhengyin Xu, Chaoying Deng, Wei Qian, Xiaobin Chen, Gongyou Chen

**Affiliations:** ^1^State Key Laboratory of Microbial Metabolism, School of Agriculture and Biology, Shanghai Jiao Tong University, Shanghai, China; ^2^State Key Laboratory of Plant Genomics, Institute of Microbiology, Chinese Academy of Sciences, Beijing, China

**Keywords:** *Xanthomonas oryzae* pv. *oryzicola*, bacterial leaf streak, two component signal transduction system (TCS), CheA, VemR, HrpG

## Abstract

Two-component systems (TCSs) (cognate sensor histidine kinase/response regulator pair, HK/RR) play a crucial role in bacterial adaptation, survival, and productive colonization. An atypical orphan single-domain RR VemR was characterized by the non-vascular pathogen *Xanthomonas oryzae* pv. *oryzicola* (*Xoc*) is known to cause bacterial leaf streak (BLS) disease in rice. *Xoc* growth and pathogenicity in rice, motility, biosynthesis of extracellular polysaccharide (EPS), and the ability to trigger HR in non-host tobacco were severely compromised in the deletion mutant strain RΔ*vemR* as compared to the wild-type strain RS105. Site-directed mutagenesis and phosphotransfer experiments revealed that the conserved aspartate (D^56^) residue within the stand-alone phosphoacceptor receiver (REC) domain is essential for phosphorelay and the regulatory activity of *Xoc* VemR. Yeast two-hybrid (Y2H) and co-immunoprecipitation (co-IP) data identified CheA as the HK co-opting the RR VemR for phosphorylation. Affinity proteomics identified several downstream VemR-interacting proteins, such as 2-oxoglutarate dehydrogenase (OGDH), DNA-binding RR SirA, flagellar basal body P-ring formation protein FlgA, Type 4a pilus retraction ATPase PilT, stress-inducible sensor HK BaeS, septum site-determining protein MinD, cytoskeletal protein CcmA, and Type III and VI secretion system proteins HrpG and Hcp, respectively. Y2H and deletion mutant analyses corroborated that VemR interacted with OGDH, SirA, FlgA, and HrpG; thus, implicating multi-layered control of diverse cellular processes including carbon metabolism, motility, and pathogenicity in the rice. Physical interaction between VemR and HrpG suggested cross-talk interaction between CheA/VemR- and HpaS/HrpG-mediated signal transduction events orchestrating the *hrp* gene expression.

## Introduction

Bacterial leaf streak (BLS) disease of rice is caused by *Xanthomonas oryzae* pv. *oryzicola* (*Xoc*) (Gram-negative *γ-Proteobacterium*) and accounts for 10–30% yield losses in the epidemic regions of South Asia (Nino-Liu et al., 2006; Bogdanove et al., 2011). *Xoc* gains entry into rice leaves through stomata and multiplies in the substomatal cavity, eventually leading to intracellular colonization of leaf parenchyma. Once the apoplastic pathogen density reaches the requisite threshold, diffusible signal factor (DSF)-mediated quorum sensing (QS) mechanisms orchestrate virulence gene expression and secretion of cell-wall degrading enzymes to breach the parenchymal cell wall and access the host nutrient pool (Büttner and Bonas, 2010; Zhou et al., 2013). BLS symptoms appear on rice leaves as small, interveinal, and water-soaked lesions that later become yellowish-gray and translucent.

Bacterial two-component regulatory systems (TCSs) play a critical role in the perception and transduction of environmental signals to ensure cellular survival, adaptability, and robust colonization of specific ecological niches. Canonical TCS regulation (sense-and-response) consists of autophosphorylation of a dimeric membrane-bound “sensor” histidine kinase (HK) in response to a specific environmental cue and subsequent phosphorelay to the conserved Asp of the cytosolic cognate “response” regulator (RR) (Jacob-Dubuisson et al., 2018). Occasionally, a hybrid sensor HK has an additional C-terminus Asp-containing domain and undergoes intramolecular transfer of the phosphoryl group from the conserved His to Asp residues. The three-step phosphorelay to a terminal RR is mediated *via* a cytosolic His phosphotransferase (Singh et al., 2021). An unusually wide array of TCS signal transduction events are arbitrated through the variability within the exposed extracellular regions of transmembrane HK sensor (input) and RR effector (output) domains (Laub and Goulian, 2007; Jung et al., 2019).

TCS-regulated fundamental physiological processes such as adaptation to osmolarity, light, temperature, and oxygen, nutrient acquisition and metabolism, antibiotic resistance, flagellar and secretion system assembly, biofilm formation, etc. are paramount for bacterial pathogenicity and virulence. Metagenomic analyses have revealed the presence of 110 TCS genes on an average, and at least 70 structurally and functionally conserved “core sets” of TCS genes across all the studied genomes of *Xanthomonas* spp. (Qian et al., 2008). Zhang et al. (2018) annotated 36 orthodox and 20 hybrid HK genes and 54 RR genes within the genome of *Xoc* strain GX01. To date, eight pairs of pathogenicity-related canonical TCSs have been characterized in *Xanthomonas* spp., namely, RpfC/G (Slater et al., 2000), RaxH/R (Burdman et al., 2004), PhoQ/P (Lee et al., 2008), ColS/R (or VgrS/R) (Wang et al., 2016), RavS/R (He et al., 2009), PdeK/R (or RavA/R) (Tao et al., 2014), HpaR2/S (Li et al., 2014), and PcrK/R (Wang et al., 2017).

Gram-negative phytopathogenic bacteria deploy injectisome Type III secretion systems (T3SSs) for the translocation of effector proteins into the host cells which in turn affect host signaling and metabolism (Alvarez-Martinez et al., 2021). The effectors also mediate host specificity and pathogenicity by triggering disease in susceptible or hypersensitive response (HR) in the resistant hosts (Jacques et al., 2016). The *Xoc* T3SS strain is encoded by the *hrp* (*h*ypersensitive *r*esponse and *p*athogenicity) gene cluster and controlled by the HrpG/HrpX regulon (Teper et al., 2021). The expression of T3SS apparatus genes and effectors is regulated by HrpX expression which is under the control of HrpG. The HrpG/HrpX regulon itself is under stringent metabolic and genetic regulation *in planta*. HrpG belongs to the OmpR family RR and perceives the environmental signals *via* its cognate sensor kinase HpaS (Li et al., 2014). The RpfC/RpfG (*r*egulation of *p*athogenicity *f*actors C and G) TCS couples the QS signaling and intracellular regulatory networks through the second messenger cyclic di-GMP and the global transcriptional regulator Crp-like protein (Clp) in *X. campestris* pv. *campestris* (*Xcc*) (Slater et al., 2000; He and Zhang, 2008). Additionally, Clp also converges environmental signals transduced by the HrpG/HrpX cascade (Wengelnik and Bonas, 1996; Wengelnik et al., 1996) thereby modulating carbohydrate utilization, cell motility, and the biosynthesis of extracellular polysaccharides (EPS) and extracellular enzymes (Guo et al., 2019).

It is axiomatic to believe that the availability, acquisition, and utilization of host cell-derived carbohydrates will have a significant impact on bacterial virulence and pathogenicity through carbon provision and regulation of biosynthesis in EPS (Guo et al., 2012, 2015, 2017, 2020; Cai et al., 2014) and DSF for QS (Zhou et al., 2015; Guo et al., 2017). Earlier work in our laboratory indicated that (a) glycolysis/gluconeogenesis enzyme fructose-bisphophate aldolase (FbaB) was required for EPS production, virulence, and growth of the *Xoc* strain RS105 in rice, and (b) *fbaB* expression was negatively and differentially regulated by both HrpG and HrpX (Guo et al., 2012). These data further confirmed that carbohydrate-mediated induction of the *hrp* gene expression is differentially regulated through discrete and differential regulation of both *hrpG* and *hrpX* (Teper et al., 2021). Regulome analyses revealed that the orphan RR VemR (and PXO_RS20790) are the master positive regulators of HrpX and execute extensive cross-talk within other pathogenicity-associated signaling networks in *X. oryzae* pv. *oryzae* (Zheng et al., 2021).

Here, we report the identification and characterization of the orphan RR protein VemR (AKO16095.1 encoded by ACU12_10515) and its cognate sensor protein CheA (Chemotaxis protein HK; AKO15518.1 encoded by ACU12_07015) from *Xoc* strain RS105 (GenBank Acc. CP011961). In flagellated bacteria, CheA/Y TCS orchestrates chemosensory adaptation to the extracellular signals resulting in biased motility. Furthermore, empirical evidence indicated that the activated VemR outputs signals to multiple interacting partners including HrpG, thereby regulating bacterial motility, EPS production, and *hrp* gene expression. CheA/VemR TCS coordinates several interconnected signaling pathways that are essential for the ability of *Xoc* to cause virulence in host rice and induce HR in non-host tobacco.

## Experimental Procedures

### Bacterial Strains, Plasmids, and Culture Conditions

All relevant strains and plasmids used in this study are listed in Supplementary Table 1. *Escherichia coli* strains were routinely grown at 37°C in the Luria–Bertani medium containing 5 g L^–1^ yeast extract, 10 g L^–1^ NaCl, 10 g L^–1^ tryptone, and 15 gL^–1^ agar. All *X. oryzae* pv. *oryzicola* (*Xoc*) strains were grown at 28°C in NB (1 g L^–1^ yeast extract, 3 g L^–1^ beef extract, 5 g L^–1^ polypeptone, 10 g L^–1^ sucrose), NA (NB with 15 g L^–1^ agar), NAN (NA without sucrose), NAS (NA with 100 g Ll^–1^ sucrose), NY (NB without beef extract and sucrose), XOM3 (1.8 g L^–1^ D-xylose, 670 μM D, L-methionine, 10 mM sodium L-glutamate, 240 μM NaFe^2+^-ethylenediaminetetraacetic acid (EDTA), 5 mM MgCl_2_, 14.7 mM KH_2_PO_4_ and 40 μM MnSO_4_; pH 6.0) (Guo et al., 2012), or NCM medium [2 g l^–1^ (NH_4_)_2_SO_4_, 4 g L^–1^ K_2_HPO_4_, 6 g l^–1^ KH_2_PO_4_, 0.2 g L^–1^ MgSO_4_⋅7H_2_O] as indicated. Antibiotics were added at the following concentrations (μg ml^–1^): kanamycin (Kan), 25; rifampicin (Rif), 50; ampicillin (Ap), 100; spectinomycin (Sp), 50, and streptomycin (Sm), 50.

### Tn*5* Mutagenesis in *X. oryzae* Pv. *oryzicola*

EZ-Tn*5*™<KAN^–1^>Tnp Transposome™ kit (Epicentre, Madison, WI, United States) was used according to Zou et al. (2011) to generate a Tn*5*-tagged transposition mutant library in the *Xoc hrpG*-deletion mutant Rδ*hrpG* expressing the *fbaBc*:*gusA* transcriptional fusion construct pfbaBcGUS (Supplementary Table 1). Single Kan-resistance colonies were individually numbered, transferred to selective NA plates with X-Gluc, and incubated for 24 h in the dark for selection of GUS-expressing phenotype. The Rδ*hrpG*:pfbaBcGUS strain was used as the positive control.

### Construction and Complementation of the Non-polar *vemR* Mutant of *X. oryzae* Pv. *oryzicola*

The non-polar mutant of *vemR* in the *Xoc* strain RS105 was constructed by using homologous recombination driven by the suicide vector, pKMS1, as previously described (Cai et al., 2014). Two flanking regions on either side of *vemR* were PCR-amplified using the primer pairs: *vemR*-upF/*vemR*-upR and *vemR*-downF/*vemR*-*down*R (Supplementary Table 2) and cloned in pMD18-T (TaKaRa, Dalian, China). Both fragments were sequence verified and subcloned into pKMS1 using *Pst*I/*Xba*I sites. The recombinant plasmid pKΔ*vemR* was introduced into the RS105 strain by electroporation and the transformants were plated on Kan-supplemented NAN plates. The single-cell colonies recovered after the first homologous recombination were cultured in NBN broth for 12 h at 28°C and then plated on NAS plates. The emergent colonies after 3–4 days were then spotted on NA alone and selective NA plates (with Kan). The eviction of the plasmid backbone after the second crossover event is indicated by Kan sensitivity yielding the required non-polar deletion mutant strain RΔ*vemR*. The fidelity of the mutagenesis protocol was independently confirmed by PCR analysis using the primer pair *vemR*-1F/*vemR*-2R. Finally, Southern hybridization (DIG; Roche Molecular Systems, Pleasanton, CA, United States) was conducted to verify the deletion of the *vemR* by using the left fragment as the probe. Other non-polar mutants of RS105 were also obtained using a similar approach.

A 681 bp DNA fragment containing the full-length *vemR* (from 300 bp upstream of the start codon to the stop codon) was PCR-amplified using the primer pair cvemR-F/cvemR-R. The sequence-verified amplicon was cloned into pML254 using *Hin*dIII/*Xba*I sites. The resulting plasmid pC*vemR* was transformed into Rδ*vemR* strain by electroporation and selected on NA plates with Kan. Fast Mutagenesis System Kit (TransGen Biotech, Beijing, China) was used for site-directed mutagenesis of *vemR*, in the complementing plasmid pC*vemR*. All the mutants were confirmed by sequencing.

### HR and Pathogenicity Assays

Hypersensitive response and pathogenicity assays were performed as described previously by Guo et al. (2012). Briefly, *Xoc* cultures were grown in selective NB broth at 28°C and 200 rpm for 16 h. Bacteria in the exponential growth phase were harvested by centrifugation, washed two times, and re-suspended in sterile water to an Abs_600_ = 0.3 (app. 1 × 10^8^CFU ml^–1^). Bacteria were then infiltrated into tobacco (*Nicotiana benthamiana*) leaves using a needless syringe and the HR was scored 24 h post-inoculation (hpi). Leaves of 2-months-old rice plants (susceptible cv. Shanyou63) were inoculated using leaf-needling for quantification of BLS lesion length 14 days-post-inoculation (dpi). All plants were maintained in the greenhouse with a 12 h day/night cycle (28/25°C day/night) and 75–80% relative humidity. Five leaves were inoculated for each independent experiment, and each experiment was repeated at least three times.

### Determination of Bacterial Growth *in Planta*

*Xoc* suspensions (1 × 10^8^CFU ml^–1^) were infiltrated into the intercellular spaces of newly expanded rice leaves (cv. Shanyou63, 2-weeks-old) with a needleless syringe at three spots on each leaf. Three 0.8 cm diameter leaf discs were excised from each infiltrated area at indicated time intervals. After being sterilized in 70% ethanol and 30% hypochlorite, the leaf discs were homogenized in 1 ml of distilled water. Diluted homogenates were plated on selective NA plates and the bacterial colonies were enumerated after 3–4 days.

### Motility Assay

*Xoc* strains were grown overnight in NB medium (Abs_600_ = 0.6) and subcultured in 20 ml NB medium for 16–18 h. The bacteria were washed two times and resuspended in sterile water (Abs_600_ = 1.0). About 2 μl of bacterial suspension was spotted on a NY plate containing 0.3% (w/v) agarose with or without 0.5% (w/v) glucose as the sole carbon source and incubated at 28°C for 2 days.

### Measurement of Extracellular Polysaccharides

**Extracellular polysaccharides** production was measured as previously described (Guo et al., 2012). Briefly, *Xoc* strains were cultured in 100 ml of NY medium alone, or NY medium supplemented with 2% (w/v) various sugars at 28°C with constant shaking at 200 rpm for 5 days. EPS was precipitated from the culture supernatant with ethanol, dried to constant weight at 55°C, and quantified.

### Quantification of Enzymatic β-Glucuronidase Activity

*Xoc* strains were precultured to mid-log phase in 5 ml NB at 28°C and subcultured (2%, v/v) in fresh 5 ml NB for 16–20 h. Bacteria were collected by centrifugation, washed two times, and resuspended (Abs_600_ = 2.0) in an *hrp*-inducing XOM3 medium (Guo et al., 2012). After incubation at 28°C for 4 h, 1 ml of the buffer containing 40 mM Tris-HCl (pH 7.0), 20 mM β-mercaptoethanol, 10 mM EDTA, and 2% Triton X-100 was added to 1 ml of the bacterial culture. The bacteria were lysed for three freeze-thaw cycles by freezing rapidly in liquid nitrogen and thawing at 37°C for 4 min. The mixture was centrifuged at 12,000 rpm for 15 min at 4°C. In total, 10 μl of supernatant was assayed for the β-glucuronidase (GUS) activity by adding 90 μl of 2 mM 4-methylumbelliferyl-β-d-glucuronide (4-MUG) (Sigma-Aldrich, Shanghai, China) and incubating at 37°C for 1 h. The reaction was terminated by adding 900 μl of 2 M Na_2_CO_3_. The GUS activity was measured at 415 nm by using the Modulus™ Single Tube Multifunction Spectrophotometer (Yuan Ping Hao, Beijing, China). One unit of enzyme activity was defined as 1 nmol of 4-methyl-umbelliferone (MU) produced min^–1^ Abs_600_^–1^ of bacterial cells.

### Real-Time Quantitative RT-PCR Analysis

The primers used for real-time quantitative RT-PCR (qRT-PCR) analysis are summarized in Supplementary Table 2. *Xoc* wild-type RS105 and RΔ*vemR* mutant strains were cultured in 20 ml NB medium for 16–20 h (Abs_600_ = 0.6), harvested by centrifugation, and washed two times before RNA extraction. Total RNA was extracted using the Trizol reagent (TaKaRa) and treated with RNase-free DNase I (TaKaRa). About 1 μg DNAse I-treated RNA was used for first-strand cDNA synthesis (RevertAid First Strand cDNA Synthesis Kit; Fermentas, Canada) in a 20 μl reaction. qRT-PCR was carried out using the Applied Biosystems™ 7500 Real-Time PCR System (Thermo Fisher Scientific, Waltham, MA, United States). The reaction mixture contained 10 μl of 2 × SYBR^®^ *Premix Ex Taq*™ (TaKaRa), 300 nM gene-specific primers, and 2 μl of 5-fold diluted cDNA template in a total volume of 20 μl. Reaction mixtures were incubated at 95°C for 30 s and cycled 40 times at 95°C for 5 s and 60°C for 34 s. The expression level of the *16S rRNA* gene was used as an internal standard. The comparative-threshold method was used to calculate the relative transcript abundance with respect to the corresponding transcript in the RS105 strain.

### *In vivo* Phosphorylation Assay

The *Xoc* RS105 *vemR* coding region was PCR-amplified using primers pET-vemR-F and pET-vemR-R. The amplicon was ligated into pMD18-T and subcloned into pET30a (Novagen, Madison, WI, United States) using the *Nde*I/*Xho*I sites. The resulting plasmid pETvemR expressing VemR with a C-terminal 6× His tag was transformed into *E. coli* strain BL21(DE3) (Invitrogen, Carlsbad, CA, United States). The overexpression and purification of pETvemR were performed as previously described (Zou et al., 2011). The plasmids pETvemR_*D*56V_, pETvemR_*D*56E_, pETcheA, and pETAtoC were constructed, overexpressed, and purified similarly.

Phos-tag SDS/PAGE was used for *in vivo* phosphorylation assays as described by Barbieri and Stock (2008). Phos-tag SDS/PAGE gels (Wako Pure Chemical Industries Ltd., Osaka, Japan) were prepared according to the manufacturer’s instructions. Briefly, SDS-PAGE gels containing 12% (w/v) 29:1 acrylamide: N,N′′-methylene-bisacrylamide, 375 mM Tris, pH 8.8, and 0.1% (w/v) SDS were copolymerized with 50 μM Phos-tag-acrylamide and 100 μM MnCl_2_. *Xoc* strains expressing C-terminus 6× His tag modified proteins were cultured in NCM medium for 36 h and total proteins were extracted. About 50 μg of total protein samples were resolved by electrophoresis at 150 V for 2 h. The SDS-PAGE gel was soaked in 150 ml transfer buffer containing 1 mM EDTA for 15 min to remove Mn^2+^ and then in 150 ml chilled transfer buffer for 15 min to remove EDTA followed by Western Blotting and detection.

### Yeast Two-Hybrid Assay

The Matchmaker™ GAL4 two-hybrid system (Clontech, Mountain View, CA, United States) was used to screen for VemR-interacting proteins. The VemR coding region was amplified and cloned into the bait vector pGBKT7 and the test genes encoding VemR-interacting protein partners were cloned into the prey vector pGADT7. The pGADT7- and pGBKT7-derived construct plasmids pB-*vemR* and pA-*X* (*X*: test gene) (Supplementary Table 1) were co-transformed into yeast (*Saccharomyces cerevisiae*) Y2H Gold cells (TaKaRa). Pairwise yeast mating was performed on yeast potato dextrose agar at 28°C for 24 h. The protein–protein interactions were detected on SD (selective minimal synthetic dropout) media. The obtained diploids were selected on DDO (Double dropout; SD/-Leu/-Trp) and QDO plates (Quadruple dropout; SD/-Leu/-Trp/-Ade/-His). Then, four 10-fold serial dilutions with sterile water were plated on QDO plates with and without 20 mg ml^–1^ 5-Bromo-4-chloro-3-indolyl-α-D-galactopyranoside (X-α-Gal). Yeast AH109 strain was co-transformed with pA-/pB-*vemR* to exclude the auto-activation ability of VemR. Co-expression of pGBKT7-*hrpF* and pGADT7-*hpa2* served as a positive interaction control (Li et al., 2011), whereas the co-expression of pGBKT7-*vemR* and pGADT7 served as the negative control.

### Tandem Affinity Purification Assay

Tandem affinity purification-vemR-F/-R primer pair was used to PCR-amplify and subclone *vemR* with tandem-linked hemagglutinin (HA; YPYDVPDYA) and FLAG (DYKDDDDK) affinity tags in the plasmid pHM1. The resulting plasmid (pH*vemR*:HA:Flag) was transformed into an Rδ*vemR* deletion mutant strain. Two 500 ml cultures of the RΔ*vemR*/pHM1 and RΔ*vemR*/pH*vemR*:HA:Flag strains were grown overnight and harvested by centrifugation. Tandem Affinity Purification (TAP) Kit (TransGen Biotech) was used for purification of FLAG- and HA-tagged VemR according to the manufacturer’s instructions. The VemR-associated proteins were resolved with SDS-PAGE and detected by silver staining. The differentially displayed protein bands were excised and prepared for liquid chromatography-mass spectrometry (LC-MS/MS) analysis (Wuli Biotech, Beijing, China).

### Co-immunoprecipitation Assay

Flag-atoc-F/-R, Flag-cheA-F/-R, and Flag-hrpG-F/-R primer pairs were used for cloning and expression of FLAG-tagged AtoC, CheA, and HrpG in pHM1 (yielding the plasmids *atoC*:Flag, *CheA*:Flag, and *hrpG*:Flag, respectively). Likewise, pET-vemR-F/-R primers were used for cloning and expression of 6× His-tagged VemR yielding the plasmid *vemR*:His. 6× His-tagged VemR expression plasmid was co-expressed with FLAG tagged-AtoC, -CheA, and -HrpG expression plasmids separately. After 12 h co-culture, total proteins were extracted with lysis buffer containing 50 mM Tris-HCl (pH 7.5), 150 mM NaCl, 1 mM EDTA, 10% (v/v) glycerol, 0.2% (v/v) Triton-X 100, 1 mM PMSF, and protease inhibitor cocktail (cOmplete™; Sigma-Aldrich). For pre-clearing, the extracted proteins were mixed with 30 μl protein A magnetic beads (Bimake, Houston, TX, United States) for 15 min at 4°C. After removing the beads, the precleared protein extract was incubated with 2.5 μg μl^–1^ anti-His antibody (TransGen Biotech) and 30 μl protein A magnetic beads for 10 h at 4°C. The beads were washed five times with washing buffer containing 50 mM Tris-HCl (pH 7.5), 300 mM NaCl, 1 mM EDTA, 10% (v/v) glycerol and 0.1% (v/v) Triton-X 100. The beads were suspended in loading buffer, boiled for 8 min, and the supernatant was used in immunoblotting assays. Proteins were separated by 10% SDS-PAGE gels and transferred to 0.2 μm PVDF membranes (Sigma-Aldrich). Anti-FLAG and anti-His antibodies (TransGen Biotech) were used for detecting VemR:6 × His and AtoC:FLAG, CheA:FLAG, and HrpG:FLAG fusions, respectively.

## Results

### *X. oryzae* Pv. *oryzicola* RR v*emR* Is Transcriptionally Repressed by HrpG

**FIGURE 1:**
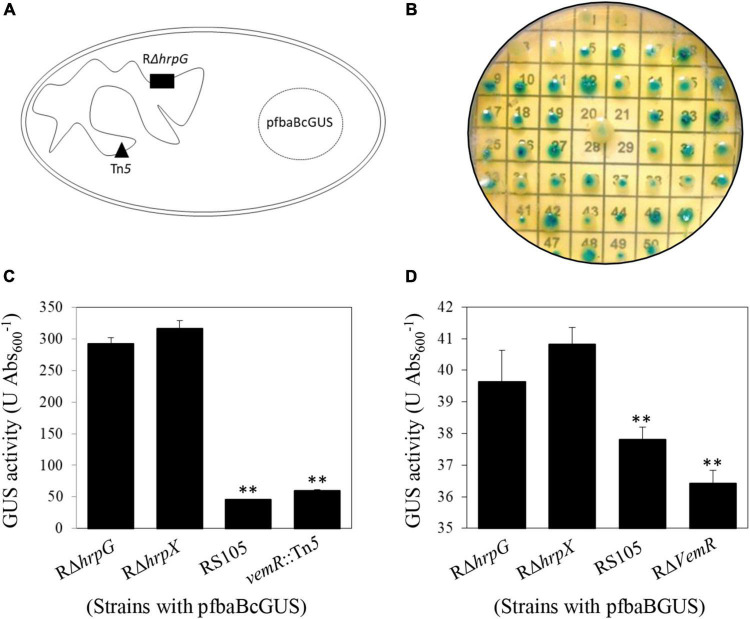
Transcriptional repression of v*emR* by HrpG in *X. oryzae* pv. *oryzicola* (*Xoc*). **(A,B)** Schematic representation of Tn*5* mutagenesis strategy in *Xoc* to identify signaling genes acting upstream of HrpG in *Xoc* strain Rρ*hrpG*/pfbaBcGUS. **(C,D)** Enzymatic GUS activity (mean ± standard deviation) in the selected Tn*5* mutant strain *vemR*:Tn*5/*pfbaBcGUS and the non-polar deletion strain Rρ*vemR*/pfbaBGUS, respectively. Student *t*-test (*p* = 0.01) was used to separate the significantly different means (denoted by **) relative to the parental Rρ*hrpG* strain used for Tn*5* mutagenesis.

To examine this regulatory link between carbohydrate acquisition, utilization, and *hrp* gene expression, we constructed a Tn*5*-tagged mutant library in a Δ*hrpG* background also carrying the episomal translational fusion reporter pfbaBcGUS (optimized PIP-box promoter of *fbaB* with a promoter-less *gusA* gene) (Figure 1A). Activity assays and sequencing identification confirmed that the GUS activity was significantly lower in the insertion mutant strain *vemR*:Tn*5* (59.64 ± 1.72 U Abs_600_^–1^) as compared to the deletion mutant strains RΔ*hrpG* (292.64 ± 9.18 U Abs_600_^–1^) or Rδ*hrpX* (316.83 ± 12.34 U Abs_600_^–1^) (Figures 1B,C). Tn*5* transposon was found to be inserted in the gene ACU12_10515 encoding the RR *vemR*.

To facilitate functional characterization of *vemR*, a non-polar deletion mutant strain RΔ*vemR* (Supplementary Table 1 and Supplementary Figure 1) was constructed by homologous suicide plasmid integration. Enzymatic GUS activity in RΔ*vemR* (36.42 ± 0.72 U Abs_600_^–1^) carrying the reporter plasmid pfbaBGUS (with the native *fbaB* promoter) was significantly lower than the RΔ*hrpG* (39.67 ± 6.71 U Abs_600_^–1^) or RΔ*hrpX* (40.83 ± 0.77 U Abs_600_^–1^) strains but similar to the wild-type RS105 strain (37.81 ± 0.64 U Abs_600_^–1^) (Figure 1D). These data precluded any polar effects of Tn*5*-insertion on VemR signaling and confirmed that *vemR* is transcriptionally repressed by HrpG in *Xoc* strain RS105.

Bioinformatic analysis revealed that *Xoc* VemR (126 aa) belongs to the NarL (Nitrate/Nitrite response regulator L) family of RRs (Katsir et al., 2015). However, ubiquitously conserved *Xanthomonas* VemR RRs contain only the stand-alone phosphoacceptor receiver (REC) domain and lack the typical helix-turn-helix (HTH) DNA-binding output domain present in *E. coli* NarL. Conserved metal-binding and putative phosphoaccepting Asp residues (D^10^D^11^ and D^56^, respectively) and active site residues, Ser (S^84^)/Phe (F^103^)^/^Lys (K^106^), participate in divalent metal ion-dependent phosphorelay reaction between the cognate HK and REC domain of the *Xoc* VemR. Indeed, the conserved D^56^ was functionally validated to be the phosphoacceptor residue (Figure 4, see below) and deemed essential for *Xoc* pathogenicity, motility, and EPS production.

**FIGURE 2:**
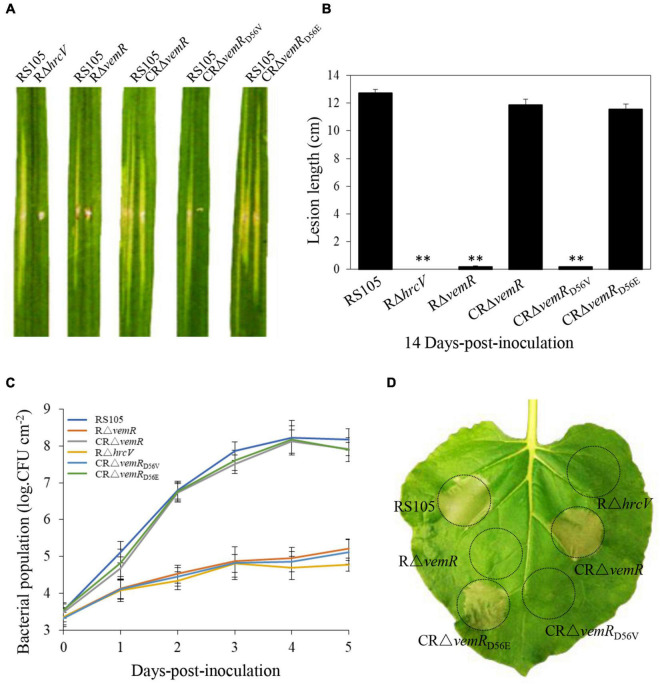
*vemR* is required for full virulence and growth of *X. oryzae* pv. *oryzicola* (*Xoc*) in rice and HR induction in tobacco. **(A)** Symptoms caused by different *Xoc* strains (suspended in water to Abs_600_ = 0.3, app. 1 × 10^8^CFU ml^– 1^) on leaves of susceptible rice cultivar Shanyou63 by leaf-needling inoculation. **(B)** Bacterial leaf streak lesions on rice leave caused by *Xoc* strains. Values are the mean ± standard deviation (SD) from three replicates, each with inoculations on five leaves. Photographs and lesion length data were taken at 14 days-post-inoculation (dpi). Student *t*-test (*p* = 0.01) was used to separate the significantly different means (denoted by **) relative to the wild-type RS105 controls. **(C)** Growth of *Xoc* mutant strains *in planta*. Bacteria were isolated from the inoculated leaf tissue excised at the indicated time points, homogenized in sterile water and the isolated bacteria were plated on selective NA plates. Bacterial colonies were enumerated after incubation at 28°C for 3 days. The presented data are the mean ± standard deviation (SD) from three independent experiments with five replicates each. **(D)** HR induction on non-host tobacco (*N. benthamiana*) leaves by *Xoc* strains (suspended in water to Abs_600_ = 0.3, app. 1 × 10^8^CFU ml^– 1^). The experiment was independently repeated three times. The photograph was taken 24 h-post-inoculation (hpi) from a representative leaf. The T3SS-deficient strain RΔ*hrcV* was used as a negative control.

**FIGURE 3:**
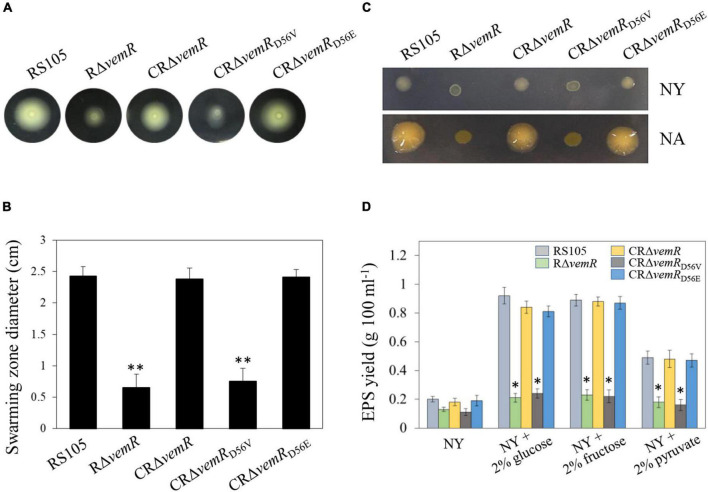
*vemR* is required for motility and EPS production in *X. oryzae* pv. *oryzicola* (*Xoc*). **(A,B)** Swarming motility of *Xoc* strains on NY medium containing 0.5% glucose. The data were scored at 2 days after inoculation. **(C)** Colony morphology and **(D)** EPS yield of *Xoc* strains in liquid NY medium and NY medium supplemented with 2% glucose, fructose, or pyruvate. Data presented are the mean ± standard deviation (SD) from three independent experiments with five replications each. Student *t*-test (*p* = 0.01 and 0.05) was used to separate significantly different means denoted by ** and *, respectively.

**FIGURE 4:**
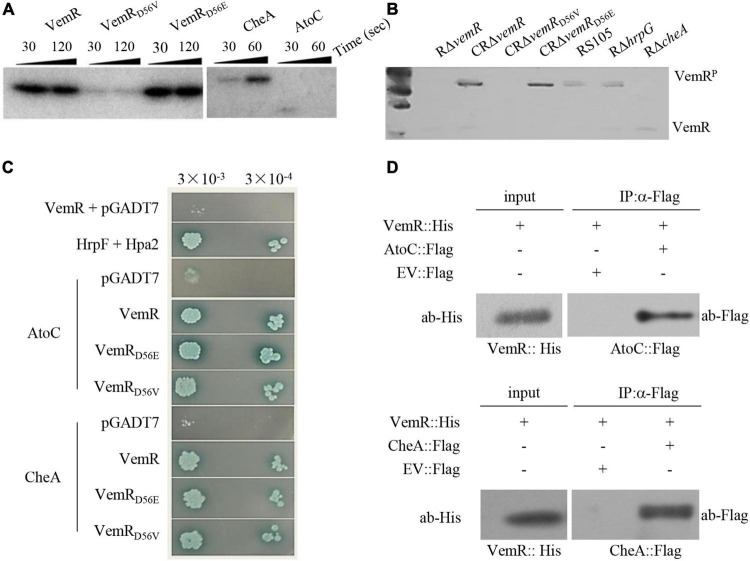
Sensor kinase CheA phosphorylates VemR in *X. oryzae* pv. *oryzicola* (*Xoc*). **(A)** *In vitro* phosphorylation assay showing phosphorylation of VemR (at D^56^ residue) and CheA. Purified VemR, VemR_*D*56E_, VemR_*D*56V_, CheA, and AtoC proteins (10 μM) were incubated with 100 μM [γ−−^32^P]ATP (10 μCi) and 10 μM histidine kinase. The reactions were stopped at the indicated time points by loading buffer prior to separation by SDS-PAGE and autoradiography. **(B)** *In vivo* assay showing phosphorylation of VemR. *Xoc* strains expressing 6× His tagged VemR proteins were cultured in MMX medium for 36 h. Total proteins from the cultures were extracted and resolved on Phos-tag acrylamide-copolymerized SDS-PAGE gel. Recombinant VemR proteins were detected using anti-VemR antiserum. **(C)** Interactions of VemR with CheA and AtoC were confirmed by yeast two-hybrid and β-gal assays. Yeast Y2H Gold cells (strain AH109) were co-transformed with the “bait” pGBKT7 and the “prey” pGADT7 derivative vectors expressing VemR fused with the GAL4 DNA binding domain (BD) and CheA or AtoC proteins fused with the GAL4 activation domain (AD), respectively. Serial dilutions of the selected co-transformants were plated on QDO (Quadruple dropout; SD/-Leu/-Trp/-Ade/-His) plates with and without X-α-Gal and incubated at 28°C for 3 days. Co-expression of pB-*hpa2*/pA-*hrpF* and pB-*vemR*/pGADT7 represent positive and negative controls, respectively. **(D)** Co-immunoprecipitation analysis showing VemR interactions with AtoC (upper panel) and CheA (lower panel). Samples before (input) and after (IP: α-His) immunopurification were analyzed by immunoblotting using anti-Flag and anti-His antibodies.

### *vemR* Is Essential for *X. oryzae* Pv. *oryzicola* Pathogenicity in Host Rice and HR Induction in Non-host Tobacco

The virulence of the deletion mutant RΔ*vemR*, the complemented CRΔ*vemR*, and the wild-type RS105 strains was tested on the susceptible hybrid rice cultivar Shangyou 63 (Figures 2A,B). The deletion mutant RΔ*vemR* failed to display any significant BLS lesions on rice (0.18 ± 0.25 cm) 14 dpi similar to the non-pathogenic T3SS mutant RΔ*hrcV* (Hartmann and Büttner, 2013), used as the negative control. By contrast, the disease lesions produced by the complemented strain CRΔ*vemR* and wild-type RS105 were compared (11.86 ± 0.41 and 12.73 ± 0.25 cm, respectively), indicating that VemR played a vital role in pathogenicity on the host rice. An explicit requirement for D^56^ phosphorylation for VemR-mediated signaling was also examined. Substitution of the conserved phosphoacceptor residue D^56^ with a similar acidic residue Glu (E) in the complementing strain CRΔ*vemR*_*D*56E_ resulted in the restoration of the disease symptoms (11.57 ± 0.38 cm) similar to the wild-type strain. However, the complemented strain CRΔ*vemR*_*D*56V_ where D^56^ was substituted with a neutral amino acid Val (V) resulted in the loss of *Xoc* pathogenicity in rice (0.17 ± 0.05 cm).

Bacterial population growth data (1 × 10^8^CFU ml^–1^ initial inoculum density) also confirmed that *vemR* was essential for *Xoc* growth *in planta* (Figure 2C). The population of the deletion mutant strain RΔ*vemR* (1.7 × 10^5^ ± 2.6 × 10^4^CFU ml^–1^) and the non-pathogenic T3SS mutant RΔ*hrcV* (6.0 ± 0.5 × 10^4^CFU ml^–1^) isolated from the inoculated rice leaves 5 dpi was significantly lower than that of the wild-type RS105 (1.5 ± 0.2 × 10^8^CFU ml^–1^) and the complemented CRΔ*vemR* (8.4 ± 0.18 × 10^8^ CFU ml^–1^) strains. Likewise, the growth of CRΔ*vemR*_*D*56E_ *in planta* was similar to the wild-type strain between 0 and 5 dpi (7.9 ± 0.33 × 10^7^ CFU ml^–1^ at 5 dpi). The inability of CRΔ*vemR*_*D*56V_ to attain the requisite population density (1.3 ± 0.25 × 10^5^ at 5 dpi) and subsequently cause disease in rice leaves confirmed that phosphorylation of D^56^ is essential for VemR-mediated signal perception and downstream transduction events.

T3SS genes are essential for *Xoc* to trigger HR on the non-host tobacco apparent at 14 hpi (Zou et al., 2011). T3SS mutant RΔ*hrcV* was used as the negative control strain to compare and examine the ability of *Xoc* VemR to trigger HR in tobacco. Consistent with the lack of pathogenicity symptoms (Figures 2A,B) and the growth curve data (Figure 2C) in rice, deletion mutant RΔ*vemR* and the complemented strain CRΔ*vemR*_*D*56V_ were ineffective in HR elicitation (Figure 2D). Taken together these data confirmed that phosphorylation of VemR (at D^56^ position) is the molecular signal required for virulence of *Xoc* in host rice and HR induction in non-host plants.

### *vemR* Regulates Motility and Extracellular Polysaccharide Production in *X. oryzae* Pv. *oryzicola*

Flagellar motility (unicellular swimming or multicellular/population migration or swarming) plays a key role in nutrient resourcing, surface probing, and subsequent colonization of host tissues by several pathogenic bacteria, including *Xanthomonas* spp. (Li et al., 2020). On NY + 0.3% (w/v) agarose plates, there was no discernible significant difference in swarming motility among the wild-type RS105, deletion mutant RΔ*vemR*, and the complemented strain CRΔ*vemR* (data not shown). However, on the NY + 0.3% agarose plates supplemented with 0.5% (w/v) glucose, the diameter of the flagellum-dependent swarming zone for the RΔ*vemR* mutant (0.65 ± 0.21 cm) was significantly smaller than that of the wild-type RS105 or the complemented strain (2.45 ± 0.15 and 2.38 ± 0.17 cm, respectively) confirming impairment of motility functions. As expected, the swarming motility of CRΔ*vemR*_*D*56V_ was significantly lower than that of CRΔ*vemR*_*D*56E_ (0.75 ± 0.21 and 2.41 ± 0.12 cm, respectively) (Figure 3A).

Extracellular polysaccharide has long been recognized as an important virulence factor contributing to *Xanthomonas* pathogenesis (Guo et al., 2020; Li et al., 2020). On the minimal NY medium plates, the colonies produced by the three pathogenic wild-type RS105, CRΔ*vemR*, and CRΔ*vemR*_*D*56E_ strains were similar in size to the non-pathogenic RΔ*vemR* and CRΔ*vemR*_*D*56V_ strains, albeit mucoid in appearance. By contrast, all three pathogenic strains (wild-type RS105, CRΔ*vemR*, and CRΔ*vemR*_*D*56E_) developed stereotypical circular, opaque, mucoid, convex, and xanthomonadin-containing yellow pigmented colonies on the sucrose-containing NA medium. Nonetheless, colonies produced by the non-pathogenic RΔ*vemR* and CRΔ*vemR*_*D*56V_ strains were significantly smaller in diameter, dry, and flat (Figure 3C).

Extracellular polysaccharides produced by various *Xoc* strains were quantified in 5-day-old cultures grown in NA, NY medium alone, and NY medium supplemented with 2% (w/v) glucose, fructose, and pyruvate (Figure 3D). *Xoc* strains showed significant differences in the EPS production when cultured in NA or NY medium alone (data not shown). Notably, the deletion mutant strain RΔ*vemR* was not compromised in growth kinetics as revealed by the population density data (CFU ml^–1^) similar to that of the wild-type RS105 and CRΔ*vemR* (data not shown). However, RΔ*vemR* produced about 60–70% less EPS than the wild-type RS105 when cultured in minimal or carbohydrate-supplemented NY media. In addition, the EPS yields of the wild-type RS105 and complemented mutant strains CRΔ*vemR* or CRΔ*vemR*_*D*56E_ were comparable, implicating a role for VemR signaling in the EPS biosynthesis by *Xoc*, thereby regulating its pathogenicity in rice.

### Sensor Histidine Kinase CheA (Chemotaxis Protein) Co-opts VemR as Its Client RR in *X. oryzae* Pv. *oryzicola*

Bioinformatic analysis revealed that *Xoc* VemR is an atypical RR with a stand-alone REC domain (Qian et al., 2008). The putative phosphoacceptor site D^56^ was functionally validated using an *in vitro* phosphorylation assay (Figure 4A). The substituted variant VemR_*D*56E_ (acidic amino acid residue as the phosphoacceptor) could be phosphorylated similar to the wild-type VemR. However, VemR_*D*56V_ with a neutral amino acid substitution could no longer accept the transfer of a phosphate group. The *in vitro* phosphorylation results were further corroborated by *in vivo* assays using Phos-tag (Figure 4B). Taken together, these data confirmed that phosphorylation of VemR (at D^56^) is a prerequisite for *Xoc* virulence in host rice (Figures 2A–C) and HR induction in non-host tobacco (Figure 2D), motility (Figures 3A,B), and EPS production (Figures 3C,D).

Simple Modular Architecture Research Tool (SMART) program and blast analysis^1^ identified a FleQ domain-containing Fis family DNA-binding transcriptional regulator AtoC (ACU12_10520-encoded AKO16096.1, 494 aa) (Supplementary Figure 2) and chemotaxis protein HK CheA (AKO15518.1, 665 aa) as the protein partners likely interacting with VemR. *E. coli* AtoS/C TCS signaling has been implicated in the regulation of numerous bacterial processes including flagellar biosynthesis (Theodorou et al., 2012). Likewise, CheA/Y TCS regulates components of chemotaxis operon involved in chemosensory adaptation to the extracellular signals perceived by methyl-accepting chemotaxis proteins (M) in *E. coli* (Muok et al., 2020).

Although yeast two-hybrid (Y2H) (Figure 4C) and co*-*immunoprecipitation (Co-IP) (Figure 4D) analyses confirmed that VemR interacted with both AtoC and CheA, the *in vitro* phosphorylation assay revealed only CheA to be a potential phosphorylation substrate (Figure 4A). *In vivo* assay using Phos-tag revealed the absence of phosphorylated VemR in the RΔ*cheA* mutant strain background, thereby confirming CheA-mediated phosphorelay to VemR (Figure 4B). These data clearly indicated that CheA is the cognate HK coopting VemR as its client for the pathogenicity of *Xoc* in rice.

### *X. oryzae* Pv. *oryzicola* VemR Outputs Signals to Multiple Targets

**FIGURE 5:**
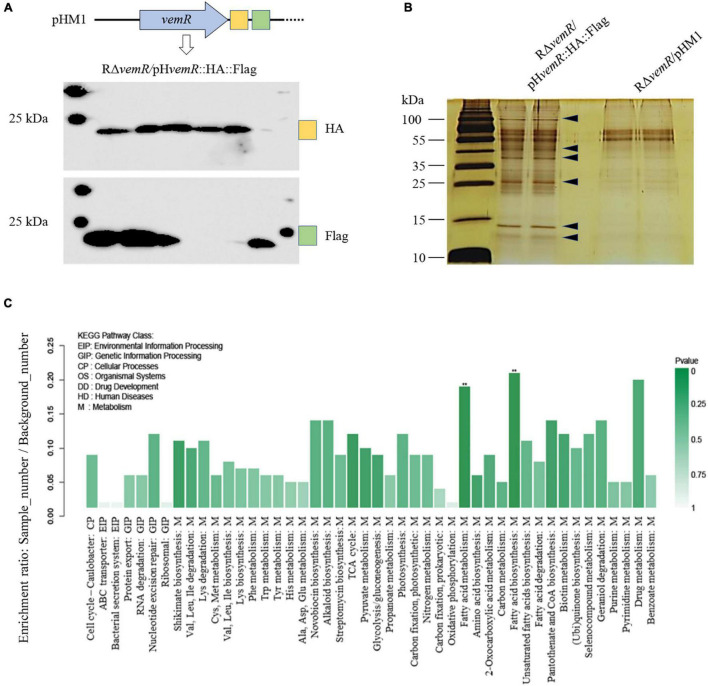
Identification of VemR-interacting protein partners in *X. oryzae* pv. *oryzicola* (*Xoc*) *via* Tandem affinity purification (TAP). **(A)** Western Blot analysis of the tandem HA- and Flag-tagged VemR expressed by the *Xoc* strain RΔ*vemR*/pH*vemR*:HA:Flag. HA and FLAG tags were detected using anti-HA and anti-Flag monoclonal antibodies. Six independent clones were analyzed for the expression of each tag. **(B)** Enriched TAP purified samples were resolved by SDS-PAGE and silver stained. *Arrows* indicated the presence of differently pulled-down proteins in presence of VemR. **(C)** Functional KEGG pathway categorization of the VemR-interacting protein partners identified *via* affinity proteomics and mass spectroscopy analyses.

VemR is an atypical RR with a stand-alone REC domain with any traditional (enzymatic or DNA-binding) output domains being absent. An affinity proteomics approach using TAP together with LC-MS/MS was therefore employed to screen potential VemR-binding proteins participating in CheA/VemR signaling cascade. For the TAP analysis, a *Xoc* strain was constructed in which the dual HA and FLAG tags were fused to the C-terminal of VemR in tandem and cloned into vector pHM1 in the Δ*vemR* background (RΔ*vemR*/pH*vemR*:HA:Flag). The antigen tags did not affect *Xoc* phenotype since the bacterial virulence on rice and the induction of the HR of the recombinant strain were similar to those of the wild-type RS105 strain (data not shown). Western blotting confirmed that the VemR-HA-FLAG was expressed *in vivo* and that both HA and FLAG monoclonal antibodies were separately immunoprecipitated (Figure 5A). Differentially expressed protein bands (Figure 5B) were excised and digested for LC-MS/MS analyses. The affinity proteomic screening detected 200 proteins that were primarily classified into four functional categories following the Kyoto Encyclopedia of Genes and Genomes (KEGG) database^2^ analyses (Figure 5C). While most of the putative VemR-binding proteins were categorized as metabolism-related, several other interacting protein partners were found to be participating in the control of the cell cycle or processing of environmental and genetic information.

VemR interaction with the enzymatic thiamine-dependent E1o component (EC 1.2.4.2) of the multimeric 2-oxoglutarate dehydrogenase (OGDH, AKO15627.1) marks the regulation of sugar and amino acid flux into tricarboxylic acid (TCA) cycle. Likewise, SirA (AKO15937.1) was also identified as a VemR-interacting DNA-binding RR that is required for homeostasis between glycolytic and gluconeogenic carbon utilization and virulence gene expression in pathogenic bacteria (Pernestig et al., 2003; Teplitski et al., 2006). Affinity proteomics also identified flagellar basal body P-ring formation protein (FlgA, AKO16075.1) and Type 4a pilus retraction ATPase (PilT, AKO15453.1) as downstream targets of CheA/VemR signaling cascade thereby influencing bacterial motility, adherence and biofilm formation (Qi et al., 2020). Finally, affinity purification of an envelope stress-inducible GAF domain-containing sensor HK BaeS (AKO17025.1) (Yu et al., 2020), septum site-determining protein MinD (WP_014502366.1) (Lutkenhaus, 2012), bactofilin family cytoskeletal protein CcmA (AKO14747.1) (Kassem et al., 2016), Type VI secretion system (T6SS) tube protein Hcp (AKO15358.1) (Alvarez-Martinez et al., 2021), and T3SS regulatory protein HrpG (Teper et al., 2021) all underscore the VemR-mediated multi-layered control of diverse but fundamental cellular processes including carbon metabolism, pathogenicity, motility, cell cycle, etc.

**FIGURE 6:**
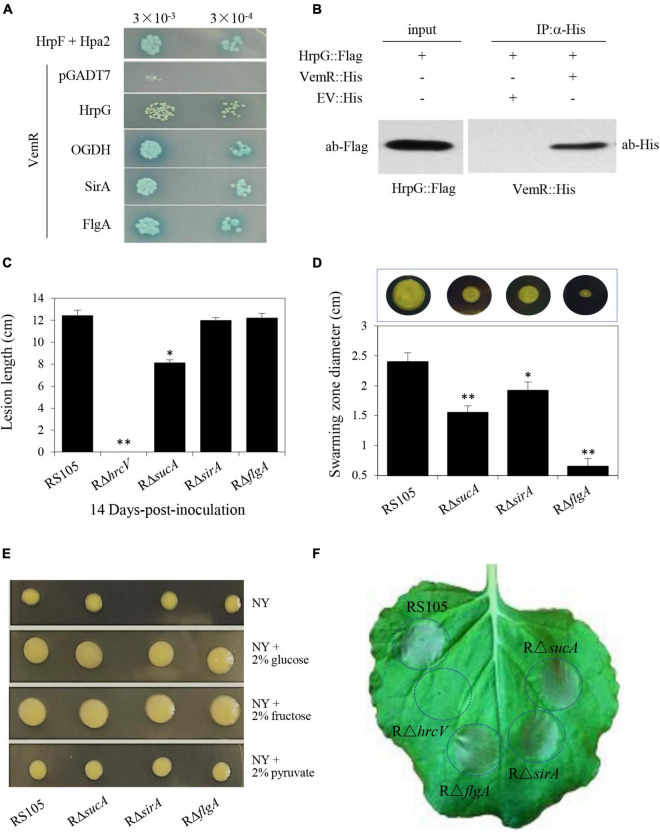
VemR interactions with multiple signaling partners differentially affect pathogenicity traits of *X. oryzae* pv. *oryzicola* (*Xoc*). **(A)** Interactions of VemR with HrpG, OGDH, SirA, and FlgA were confirmed by yeast two-hybrid and β-gal assays. Yeast Y2H Gold cells (strain AH109) were co-transformed with pGBKT7-derived “bait” and pGADT7-derived “prey” vectors expressing VemR fused with the GAL4 DNA binding domain (BD) and test proteins fused with the GAL4 activation domain (AD), respectively. Serial dilutions of the selected transformants were plated on QDO (Quadruple dropout; SD/-Leu/-Trp/-Ade/-His) plates with and without X-α-Gal and incubated at 28°C for 3 days. Co-expression of pB-*hpa2*/pA-*hrpF* and pB-*vemR*/pGADT7 represent positive and negative controls, respectively. **(B)** Co-immunoprecipitation analysis showing VemR interactions with HrpG. Samples before (input) and after (IP:α-His) immunopurification were analyzed by immunoblotting using anti-Flag and anti-His antibodies. **(C)** Bacterial leaf streak lesions on rice leave caused by *Xoc* deletion mutant strains Rρ*sucA*, Rρ*sirA*, Rρ*flgA*, and Rρ*hrcV* (T3SS-deficient strain, used as the negative control) 14 days-post-inoculation (dpi). **(D)** Swarming motility of *Xoc* strains Rρ*sucA*, Rρ*sirA*, Rρ*flgA* on NY medium containing 0.5% glucose. The data were scored 2 days after-inoculation. **(E)** Colony morphology of *Xoc* strains Rρ*sucA*, Rρ*sirA*, Rρ*flgA* as compared to the wild-type RS105 on NY medium with 2% glucose, fructose, or pyruvate. **(F)** HR induction on non-host tobacco (*N. benthamiana*) leaves by *Xoc* strains Rρ*sucA*, Rρ*sirA*, Rρ*flgA*, and Rρ*hrcV*. The experiment was independently repeated three times. The photograph was taken 24 h post-inoculation (hpi) from a representative leaf. Values presented are the mean ± standard deviation (SD) from three independent experiments with five replications each. Student *t*-test (*p* = 0.01 and 0.05) was used to separate significantly different means denoted by ** and *, respectively.

Interactions of HrpG, OGDH, SirA, and FlgA with VemR were further corroborated by Y2H data presented in Figure 6A. The physical binding of VemR and HrpG was also confirmed by co-IP (Figure 6B). The virulence of the *Xoc* deletion mutant strains RΔ*sucA* (ACU12_07715 encoding OGDH subunit E1o), RΔ*sirA* (ACU12_09595), and RΔ*flgA* (ACU12_10415) was examined (Figure 6C). The pathogenicity of RΔ*sucA* on rice was only partially reduced (lesion length 8.13 ± 0.26 cm). However, RΔ*sirA* and RΔ*flgA* displayed significant BLS lesions on rice (11.99 ± 0.24 cm and 12.22 ± 0.42 cm, respectively) similar to the wild-type strain RS105 (12.43 ± 0.48 cm). Additionally, the three deletion mutant strains, RΔ*sucA*, RΔ*sirA*, and RΔ*flgA*, displayed compromised motility (1.55 ± 0.13, 1.92 ± 0.14, and 0.65 ± 0.11 cm, respectively) as compared to the wild-type strain (2.4 ± 0.15 cm) (Figure 6D). However, any significant differences in the EPS production (Figure 6E) and ability to trigger HR in tobacco by RΔ*sucA*, RΔ*sirA*, and RΔ*flgA* were not evident (Figure 6F). These data clearly indicated precise molecular cross-talk and coordination between divergent CheA/VemR signal transduction outputs affecting bacterial virulence, motility, and EPS production.

### VemR/HrpG Interaction Regulates the Expression of HrpG Regulon in *X. oryzae* Pv. *oryzicola*

**FIGURE 7:**
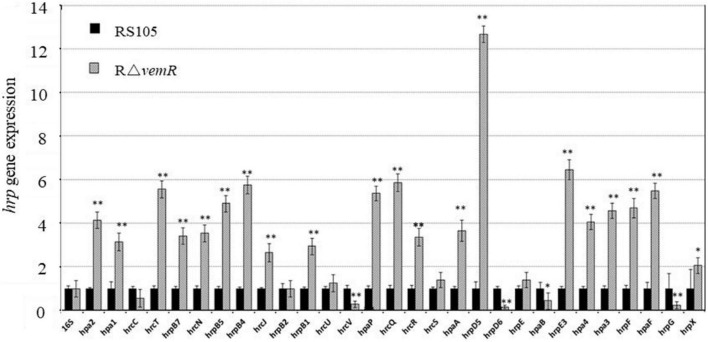
*hrp* gene expression in *X. oryzae* pv. *oryzicola* (*Xoc*) is regulated by *vemR*. *Xoc* wild-type RS105 and the deletion mutant strain RΔ*vemR* were induced in XOM3 medium for the expression analysis of the *hrp* genes. The comparative-threshold method was used to calculate the relative transcript abundance with respect to the corresponding transcript in the RS105 strain. The expression level of the *16S rRNA* gene was used as an internal control. Student *t*-test (*p* = 0.01 and 0.05) was used to separate significantly different means denoted by ** and *, respectively.

The HpaS/HrpG TCS regulates the expression of the *hrp* gene cluster *in planta* (Li et al., 2014). The expression of *fbaB* was negatively regulated by the *hrp* regulatory genes *hrpG* and *hrpX* (Guo et al., 2012); whereas the *vemR* mutation resulted in the reduced *fbaB* expression (Figures 1C,D). These results prompted us to examine the VemR-mediated regulation of *hrp* genes. Although HpaS is the cognate HK for HrpG (Li et al., 2014), co-immunoprecipitation of HrpG with VemR (Figure 6B) suggested cross-talk between CheA/VemR- and HpaS/HrpG-mediated signal transduction events orchestrating the *hrp* gene expression. Quantitative RT-PCR analysis data are summarized in Figure 7 and show the transcriptional repression of DNA-binding RR *hrpG* and T3SS proteins encoded by *hrpD6* and *hrcV* in the RΔ*vemR* mutant. By contrast, several other *hrp* genes such as *hpa2*, *hrcT*, *hrpB4*, and *hrpD5* were transcriptionally upregulated in the RΔ*vemR* mutant as compared to the wild-type RS105. However, the expression levels of *hrcC*, *hrpB2*, *hrcU*, *hrcS*, and *hrpE* were similar in both RΔ*vemR* mutant and wild-type strains.

## Discussion

The exceptionally broad host range, tissue specificity, and virulence of *Xanthomonas* spp. is well-documented (Jacques et al., 2016; An et al., 2020; Timilsina et al., 2020).). Molecular determinants of virulence are regulated in response to niche-specific extra- and intercellular signals perceived and transduced by the bacterial TCSs (Zheng et al., 2021). A typical TCS signaling cascade entails activation of a sensor HK *via cis*- or *trans*-autophosphorylation and subsequent phosphorelay to a cognate RR. Archetypical RRs always comprise a phosphoacceptor REC domain and the majority may harbor additional output effector domains with DNA-, RNA- or protein-binding or enzymatic activities (Jacob-Dubuisson et al., 2018).

In *Xoc*, FbaB activity is required for bacterial growth in presence of pyruvate, malate, and fructose, EPS production which leads to virulence in rice. Stronger inhibition of *fbaB* expression by HrpG than by HrpX also indicated differential regulation of carbohydrate utilization by the *hrp* regulon *in planta* (Guo et al., 2012). A pfbaBcGUS reporter was therefore used in the RΔ*hrpG* background to enable the identification of a Tn*5*-inserted mutant (*vemR*:Tn*5*) that likely acted upstream of the *hrpG* signaling cascade leading to reduced GUS expression (Figure 1). Bioinformatic analyses revealed that *vemR* encoded an atypical RR with a stand-alone REC-domain architecture that is ubiquitously conserved within the *Xanthomonas* spp. and lacks any conventional output effector domains. A similar, single-domain McvR (motility, chemotaxis, and virulence-related) RR involved in motility, chemotaxis, and virulence was recently identified in *Xcc* (Li et al., 2022). Such single-domain RRs have been proposed to facilitate crosstalk, feedback control, and long-range communication among members of the TCS networks (Paul et al., 2008).

The chemotaxis sensor kinase CheA was identified as the HK coopting and phosphorylating the orphan RR VemR at the D56 residue position in *Xoc* (Figure 4B). CheA/VemR signaling had a significant bearing on *Xoc* virulence and growth in rice, motility, EPS production, and ability to cause HR in non-host tobacco (Figures 2, 3). The cytosolic sensor HK CheA, the transmembrane MCPs, and the cytoplasmic adaptor protein (CheW) together form the supramolecular chemosensory lattice that is central to the directional flagellar motility in bacteria (Muok et al., 2020). *X. oryzae* pv. *oryzae* (*Xoo*) chemotaxis and motility components were essential for virulence and their expression was coordinately upregulated *in planta* (Kumar Verma et al., 2018). It is apparent that CheA/VemR signaling provides a discernible regulatory node linking chemotactic motility, EPS biosynthesis, and *Xoc* pathogenicity determinants in response to extracellular carbohydrate milieux. VemR has been shown to function as the RpoN2 cognate activator to positively regulate the transcription of the flagellar rod gene *flgG* in *X. citri* ssp. *citri* (Wu et al., 2019). Several paralogs encoding core chemotaxis signaling components annotated in the *Xoo* genome are regulated, albeit differentially, by the alternative σ^54^ factors RpoN1 and RpoN2 (Yu et al., 2021).

*rpoN*, *vemR*, and *atoC* are organized in a transcriptional operon in *Xoc* (Supplementary Figure 1), whereas *vemR* is flanked by the *rpoN2* and *fleQ* genes in *Xcc* (Tao and He, 2010). In *Xcc*, *fleQ* acts epistatic to the *vemR* gene, and FleQ functions downstream of VemR in the flagellar signaling pathway (Tao and He, 2010). *Xoc* VemR also interacted (however, sans any phosphorelay) with AtoC, another well-characterized RR (Figure 4). *E. coli* AtoS/C TCS regulates the expression of flagellar biosynthesis genes in *E. coli* (Theodorou et al., 2012). Structural feature interrogation^3^ within *Xoc* AtoC revealed presence of FleQ (aa positions 5–121), σ^54^-interaction (aa positions 139–305), AAA-ATPase (aa positions 158–305), and helix-turn-helix (HTH) (aa positions 442–483) domains. Based on gene-neighborhood and domain conservation criteria, it is likely that *Xoc* AtoC functions in a manner similar to *Xcc* FleQ. Hierarchical control of flagellin FliC expression in *Xcc* is mediated through coordinated expression of *rpoN2*-encoded σ^54^ and *fleQ*-encoded cognate σ^54^ activator directing the expression of a second alternative σ factor (σ^28^) FliA (Yang et al., 2009). Although insertional inactivation of the *fleQ* gene resulted in impaired motility and virulence in the *Xcc* strain XCP20 (Yang et al., 2009), *fleQ* mutation showed fewer phenotypic defects in *Xcc* strain 8004 (Tao and He, 2010). Likewise, both insertional inactivation and in-frame deletion of the *atoC* gene had no significant impairment of virulence, motility, and EPS production in *Xoc* RS105 (data not shown).

Affinity proteomics identified multiple VemR-interacting partners as its downstream targets (Figures 5, 6). These data conform to a fundamental regulatory role of VemR as a signal transduction node for diversifying cellular responses to CheA-mediated sensing of extracellular signals. A differential functional role of OGDH, SirA, and FlgA mediating downstream VemR signaling was evident through partially to fully reduced BLS symptoms on rice (Figure 6C) and compromised swarming motility (Figure 6D) in RΔ*sucA*, RΔ*sirA*, and RΔ*flgA* deletion mutants. However, the lack of any discernible effects of these mutations on EPS biosynthesis and the ability to cause HR on non-host tobacco warrants further investigation (Figures 6E,F).

VemR interaction with the TCA cycle enzyme OGDH and DNA-binding RR SirA provides a functional link between carbon acquisition and utilization through modulating TCA flux, glycolytic and gluconeogenic pathways (Pernestig et al., 2003). Orthologs of the *Salmonella* BarA/SirA TCS are required for virulence, motility, secondary metabolism, and stress survival throughout the γ-proteobacteria (Teplitski et al., 2006). VemR interaction with the BaeS sensor HK implicated its role in the Bae (bacterial adaptive response) stress response. BaeS/R TCS activity upregulates the expression of periplasmic chaperon proteins and several efflux pumps in response to any unfavorable molecular exposure including antibacterial agents, detergents, heavy metals, etc. (Yu et al., 2020).

It is plausible that CheA/VemR signaling also participates in regulating cell division in response to the prevalent environmental conditions such as carbohydrate availability. VemR interactions with MinD (for Z-ring positioning at mid-cell during cell division) (Lutkenhaus, 2012) and bactofilin family cytoskeletal protein CcmA (for cell shape regulation) (Kassem et al., 2016) lends further credence to above speculation. Notably, the ubiquitous family of MinD proteins is also instrumental in the correct spatiotemporal placement of macromolecular assemblies such as flagella, pili, and chemosensory apparatus (Nußbaum et al., 2020). VemR signaling also bears significantly on flagellar swimming, pilus-dependent twitching motility, and biofilm adhesion by virtue of its interactions with flagellar basal body P-ring formation protein FlgA and pilus retraction ATPase PilT (Qi et al., 2020).

Evidence suggests that HrpG is also an orphan response regulator like VemR (Li et al., 2014). VemR/HrpG interactions are therefore noteworthy (Figures 6A,B) and underline the hierarchical regulatory role of VemR within the intersecting signaling network of pathogenicity-associated regulators where HrpX is the final executant (Han et al., 2019; Zheng et al., 2021). Stimulated by the host plant environment, the RR HrpG is activated by its cognate HK (HpaS in *Xcc*) and regulates the expression of key transcriptional regulator *hrpX* and other *hrp* genes (Li et al., 2014). VemR/HrpG interaction implies that the *vemR* mutation was unable to activate HrpG thereby compromising the expression of several *hrp* components for the T3SS (Figure 7). It is conceivable that the differential regulation of various *hrp* genes in the RΔ*vemR* background causes loss of structural and/or functional integrity of T3SS in *Xoc* thereby compromising its pathogenicity.

Canonical TCSs function as cognate HK/RR pairs and the encoding genes are often co-transcribed to form an operon. Nonetheless, “many-to-one” or “one-to-many” branched TCS phosphorelay pathways have also been well-described (Francis and Porter, 2019). More than one phosphorylation sources or targets within the branched TCS networks contextually integrate multiple stimuli and/or diversify the cellular responses. For instance, the sensor HK HpaS can phosphorylate several RRs such as HrpG (Li et al., 2014), HpaR2 (Li et al., 2014), and VemR (Li et al., 2020) in *Xcc.* Likewise, orphan RR VemR can also be co-opted by RavA (Lin et al., 2022) in *Xcc* and by CheA in *Xoc* (this research) thereby differentially regulating motility, EPS biosynthesis, and virulence. Similarly, sensor kinase CheA phosphorylates two RRs CheY and CheB in *E. coli* (Szurmant and Ordal, 2004) and five RRs CheB1/B2/Y3/Y4/Y6 and CheA3 in *Rhodobacter sphaeroides* (Scott et al., 2010).

CheA kinase activity transduces the chemotaxis signal perceived *via* transmembrane chemoreceptors resulting in biased and directional flagellar motility of bacteria toward a favorable environment (Muok et al., 2020). However, the precise molecular nature of upstream activation of the CheA/VemR signaling cascade in *Xoc* is currently unknown. *Xanthomonas* spp. are endowed with a large set of TCS-encoding genes (Qian et al., 2008; Zhang et al., 2018) and high-fidelity molecular cross-talk between these TCS signaling cascades (Han et al., 2019; Kim et al., 2019) underscores its notoriety to adapt and successfully infect an exceptionally wide range of plant species.

## Data Availability Statement

The original contributions presented in the study are included in the article/Supplementary Material, further inquiries can be directed to the corresponding author.

## Author Contributions

LC and WM performed the research. LC, LZ, XX, ZX, CD, WQ, XC, and GC analyzed the data. LC, WM, and GC wrote the manuscript. GC designed the research and provided funding support. All authors read and approved the final manuscript.

## Conflict of Interest

The authors declare that the research was conducted in the absence of any commercial or financial relationships that could be construed as a potential conflict of interest.

## Publisher’s Note

All claims expressed in this article are solely those of the authors and do not necessarily represent those of their affiliated organizations, or those of the publisher, the editors and the reviewers. Any product that may be evaluated in this article, or claim that may be made by its manufacturer, is not guaranteed or endorsed by the publisher.

## Footnotes

1
http://smart.embl-heidelberg.de/

2
https://www.genome.jp/kegg/

3
https://version11.string-db.org

## Supplementary Material

Supplementary Figure 1 Schematic representation for the construction of non-polar deletion mutant strain Rρ*vemR* in *X. oryzae* pv. *oryzicola via* double homologous crossover events. The primer pairs used for the verification of the deletion event are appropriately indicated.

Supplementary Figure 2 Putative VemR interactions in *X. oryzae* pv. *oryzicola* identified *via* Simple Modular Architecture Research Tool (SMART) analysis.
