# Supplementary figures and images for: Xanthomonas oryzae Pv. oryzicola Response Regulator VemR Is Co-opted by the Sensor Kinase CheA for Phosphorylation of Multiple Pathogenicity-Related Targets
Source: Front Microbiol. 2022 Jun 9;13:928551. doi: 10.3389/fmicb.2022.928551 (PMC9218911; doi:10.3389/fmicb.2022.928551)

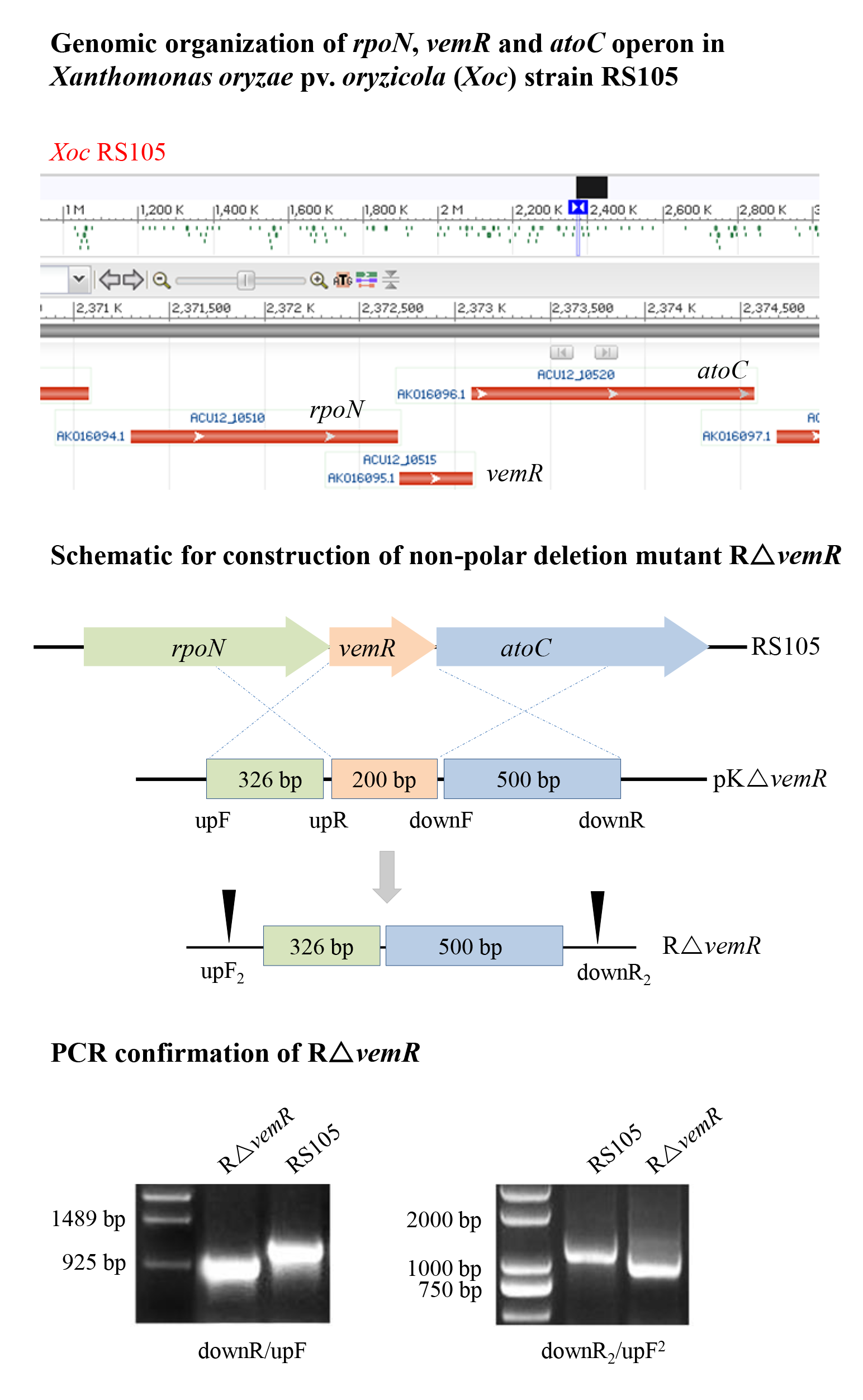

Supplement: Supplementary Figure 1 — Schematic representation for the construction of non-polar deletion mutant strain RρvemR in X. oryzae pv. oryzicola via double homologous crossover events. The primer pairs used for the verification of the deletion event are appropriately indicated. [file Image_1.TIF]

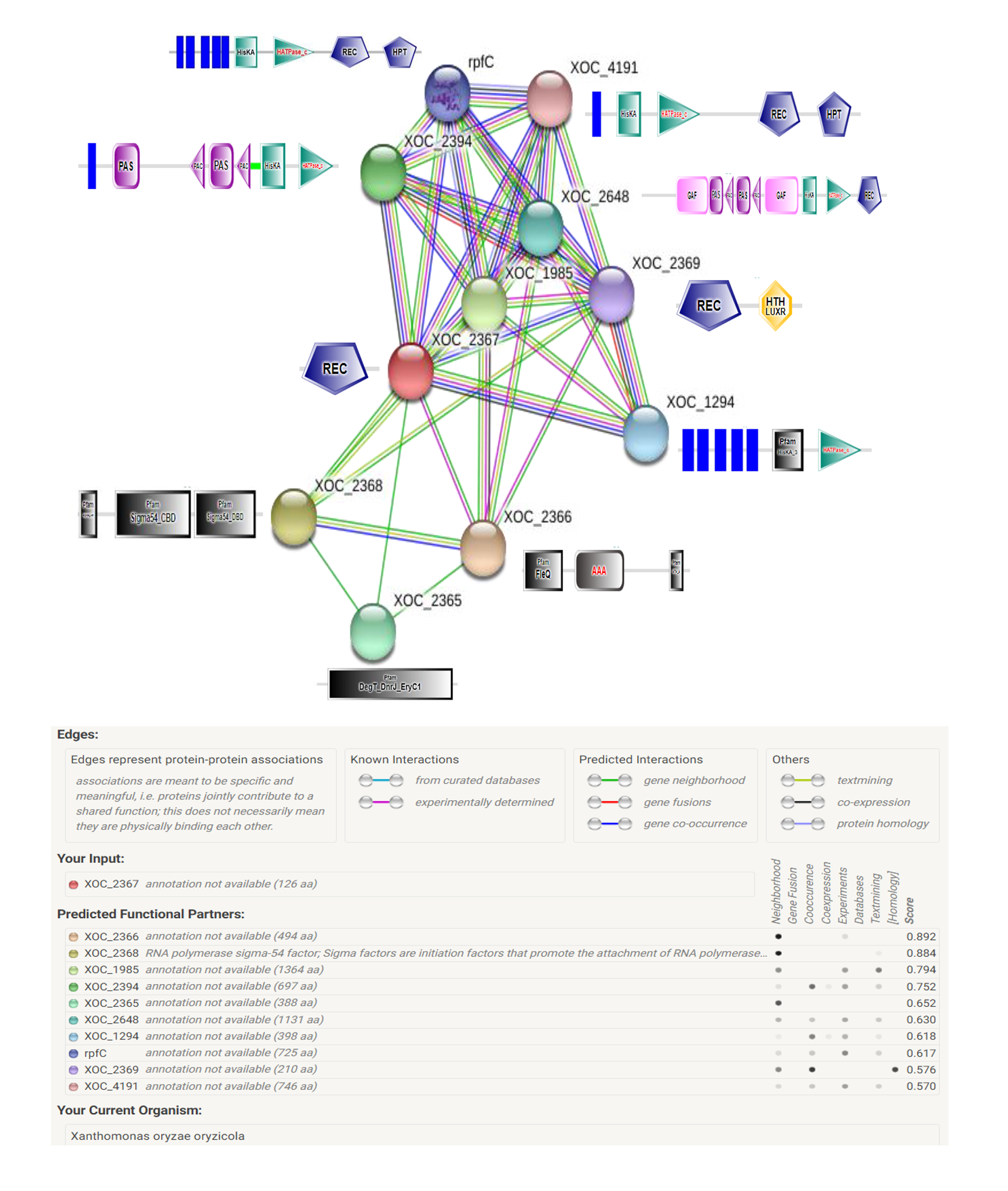

Supplement: Supplementary Figure 2 — Putative VemR interactions in X. oryzae pv. oryzicola identified via Simple Modular Architecture Research Tool (SMART) analysis. [file Image_2.TIF]
