# Supplementary material for: Xanthomonas oryzae Pv. oryzicola Response Regulator VemR Is Co-opted by the Sensor Kinase CheA for Phosphorylation of Multiple Pathogenicity-Related Targets
Source: Front Microbiol. 2022 Jun 9;13:928551. doi: 10.3389/fmicb.2022.928551 (PMC9218911; doi:10.3389/fmicb.2022.928551)
Supplement: Supplementary file 3 [file Table_1.DOCX]

**Supplementary Table 1** Bacterial strains and plasmids used in this study.

|  |  | |  |
| --- | --- | --- | --- |
| **Strains / plasmids** | **Relevant characteristics^a^** | **Reference / source** | |
|  |  | |  |
|  |  | |  |
| *Xanthomonas oryzae* pv. *oryzicola* | | | |
|  |  | |  |
| RS105 | Wild-type, causal agent of bacterial leaf streak in rice, Rif^R^ | | Zou et al., 2006 |
| R*∆fbaB* | *fbaB* deletion mutant of RS105, Rif^R^ | | Guo et al., 2012 |
| R∆*hrpG* | *hrpG* deletion mutant of RS105, Rif^R^ | | Guo et al., 2012 |
| R∆*hrpX* | *hrpX* deletion mutant of RS105, Rif^R^ | | Guo et al., 2012 |
| R∆*hrpV* | *hrpV* deletion mutant of RS105, Rif^R^ | | Guo et al., 2012 |
| R∆*vemR* | RS105 containing a 200-bp deletion in *vemR*, Rif^R^ | | This study |
| CR∆*vemR* | R∆*vemR* containing pHvemR *in trans,* Rif^R^, Sp^R^ | | This study |
| CR∆*vemR_D56E_* | R∆*vemR* containing pHvemR_D56E_ *in trans,* Rif^R^, Sp^R^ | | This study |
| CR∆*vemR_D56V_* | R∆*vemR* containing pHvemR_D56V_ *in trans,* Rif^R^, Sp^R^ | |  |
| RS105/Tn*5*::*vemR* | RS105 containing Tn*5* insertion in *vemR,* Rif^R^, Kan^R^ | | This study |
| R∆*vemR*/pH*vemR*::HA::Flag | R∆*vemR* containing pH*vemR*::HA::Flag, Rif^R^, Sp^R^ | | This study |
| R∆*vemR*/pHM1 | ∆*vemR* containing pHM1 plasmid, Rif^R^, Sp^R^ | | This study |
| *vemR*::His | RS105 containing pET*vemR* with His tag, Rif^R^, Kan^R^ | | This study |
| *atoC*::Flag | RS105 containing pH*atoC* with Flag tag, Rif^R^, Sp^R^ | | This study |
| *cheA*::Flag | RS105 containing pH*cheA* with Flag tag, Rif^R^, Sp^R^ | | This study |
| *hrpG*::Flag | RS105 containing pH*hrpG* with Flag tag, Rif^R^, Sp^R^ | | This study |
|  |  | |  |
| *Escherichia coli* | | | |
|  |  | |  |
| TOP10 | F^–^*mcr*A Δ(*mrr*-*hsd*RMS-*mcr*BC) φ80*lac*ZΔM15 Δ*lac*X74 *rec*A1 *ara*D139 Δ(*ara-leu*)7697 *gal*U *gal*K λ^–^*rps*L(Str^R^) *end*A1 *nup*G | | Invitrogen |
| DH5α | F^–^ φ80*lac*ZΔM15 Δ(*lac*ZYA-*arg*F)U169 *rec*A1 *end*A1 *hsd*R17(r_K_^–^, m_K_^+^) *pho*A *sup*E44 λ-thi-1 *gyr*A96 *rel*A1 | | Clontech |
| BL21 (DE3) | F^–^*omp*T *hsd*S_B_ (r_B_^–^, m_B_^–^) *gal dcm* (DE3) | | Novagen |
|  |  | |  |
| *Saccharomyces cerevisiae* | | | |
|  |  | |  |
| AH109 | *MATa trp1-901 leu2-3 112 ura3-52 his3-200 gal4 gal80 LYS2::GAL1_UAS_-GAL1_TATA_-His3* | | Clontech |
|  |  | |  |
| Plasmids |  | |  |
|  |  | |  |
| pMD19-T | pUC origin, cloning vector, Ap^R^ | | TaKaRa |
| pHM1 | Broad-host range cosmid, *parA IncW* derivative of pRI40, Sp^R^, Sm^R^ | | Guo et al., 2012 |
| pH*vemR* | 681-bp *vemR* and upstream region in pHM1 | | This study |
| pKMS1 | Suicide vector derived from pK18mobGII, *sacB^+^*, Kan^R^ | | Zou et al., 2011 |
| pET30a(+) | pBR322 origin, *lacI,* His-tag at C-terminus, Kan^R^ | | Novagen |
| pKΔ*vemR* | Contains fusion of the left (326-bp) and right (500-bp) regions flanking *vemR* (200 bp) in pKMS1, Kan^R^ | | This study |
| pGBKT7 | Bait domain of GAL4, c-myc epitope tag, *TRP1*, Kan^R^ | | TaKaRa |
| pGADT7 | Activation domain of GAL4, HA epitope tag, *LEU2*, Ap^R^ | | TaKaRa |
| pB-*vemR* | *vemR* in pGBKT7, Kan^R^ | | This study |
| pB-*vemR*_D56E_ | *vemR_D56E_* in pGBKT7, Kan^R^ | | This study |
| pB-*vemR*_D56V_ | *vemR_D56V_* in pGBKT7, Kan^R^ | | This study |
| pB-*hpa2* | *hpa2* in pGBKT7, Kan^R^ | | Zou et al., 2011 |
| pA-*atoC* | *atoC* in pGADT7, Ap^R^ | | This study |
| pA-*cheA* | *cheA* in pGADT7, Ap^R^ | | This study |
| pA-*hrpF* | *hrpF* in pGADT7, Ap^R^ | | Zou et al., 2011 |
| pA-*sucA* | *sucA* in pGADT7, Ap^R^ | | This study |
| pA-*sirA* | *sirA* in pGADT7, Ap^R^ | | This study |
| pA-*flgA* | *flgA* in pGADT7, Ap^R^ | | This study |
| pfbaBcGUS | Contains the *fbaB* promoter fused to glucuronidase, Kan^R^ | | Guo et al., 2012 |
|  |  | |  |

^a^Ap, ampicillin; Kan, kanamycin; Rif, rifampicin; Sm, streptomycin; and Sp, spectinomycin.

**References**

Guo W., Zou L. F., Li Y. R., Cui Y. P., Ji Z. Y., et al. (2012). Fructose-bisphophate aldolase exhibits functional roles between carbon metabolism and the *hrp* system in rice pathogen *Xanthomonas oryzae* pv. *oryzicola*. *PLoS One,* 7.

Zou L. F., Li Y. R., Chen G. Y. (2011). A non-marker mutagenesis strategy to generate poly-*hrp* gene mutants in the rice pathogen *Xanthomonas oryzae* pv. *oryzicola*. *Agricult. Sci. China* 10, 1139-1150.

Zou L. F., Wang X. P., Xiang Y., Zhang B., Li Y. R., et al. (2006.) Elucidation of the *hrp* cluster of *Xanthomonas oryzae* pv. *oryzicola* that controls the hypersensitive response in nonhost tobacco and pathogenicity in susceptible host rice. *Appl. Environ. Microbiol.* 72, 6212-6224.
