# Supplementary material for: Xanthomonas oryzae Pv. oryzicola Response Regulator VemR Is Co-opted by the Sensor Kinase CheA for Phosphorylation of Multiple Pathogenicity-Related Targets
Source: Front Microbiol. 2022 Jun 9;13:928551. doi: 10.3389/fmicb.2022.928551 (PMC9218911; doi:10.3389/fmicb.2022.928551)
Supplement: Supplementary file 4 [file Table_2.DOCX]

**Supplementary Table 2:** List of primers used in this study.

|  |  |  |
| --- | --- | --- |
| **Primer** | **Sequence (5′→3′) (restriction sites underlined)** | **Description** |
|  |  |  |
|  |  |  |
| Tn*5*-F | AGATGTGTATAAGAGACAG | Verifying EZ-Tn*5* insertion site |
| Tn*5*-R | CTGTCTCTTATACACATCT |  |
| vemR-up-F | TATCTGCAGGCAGGGGGGTGTCCAATGCCCAGAC | 500-bp fragment on right side of *vemR* for deletion mutagenesis |
| vemR-up-R | GCGGATCCCACGCGTCTGATCAAGACGCAGGAC |  |
| vemR-downF | CGGGATCCAGTAGTCCTTATCCTGCCCGGGCAA | 326-bp fragment on left side of *vemR* for deletion mutagenesis |
| vemR-downR | ACGTCTAGAGAATCGACGATCTCGCGCATCACCA |  |
| vemR-upF2 | GCAGGGGGGTGTCCAATGCCCAGAC | 1026-bp fragment containing right and left border of *vemR* |
| vemR-downR2 | GAATCGACGATCTCGCGCATCACCA |  |
| cvemR-F | CGAGCTCATGAGCAAACTCACCGTGCTGCTGG | 681-bp fragment containing full-length *vemR* gene and promoter region |
| cvemR-R | GGGGTACCTTCCTGGCTCCTTACCGTCAGGCCC |  |
| pB-vemR-F | CGGAATTCATGAGCAAACTCACCGTGCTGCTGG | 381-bp *vemR* fragment for cloning in pGBKT7 |
| pB-vemR-R | CGGGATCCATCATTCCTGGCTCCTTACCGTCAGG |  |
| pET-vemR-F | CGCCATATGATGAGCAAACTCACCGTGCTGCTGG | for cloning full-length *vemR* in pET30a |
| pET-vemR-R | CCGCTCGAGTTCCTGGCTCCTTACCGTCAGGCCC |  |
| vemRD56V-F | CAATGTCGTGCTGATGGTCCTTGCC | for cloning *vemR*_D56V_ in pET30a |
| vemRD56V-R | CCATCTCCGGCATGGCAAGGACCAT |  |
| vemRD56E-F | CAATGTCGTGCTGATGGAACTTGCC | for cloning *vemR*_D56E_ in pET30a |
| vemRD56E-R | CCATCTCCGGCATGGCAAGTTCCAT |  |
| pA-atoC-F | CGCCATATGATGAGTGAGTCCCGCATTCTGTTGA | for cloning 1485-bp *atoC* in pGADT7 |
| pA-atoC -R | CCGCTCGAGGTTGGCCAGCTCGGTCTGCTCGCGG |  |
| pA-cheA-F | ATTCCATATGATGGACATGAACCAGCTGAT | for cloning 1998-bp *cheA* in pGADT7 |
| pA-cheA-R | TCCCCCGGGAATCAGGCCGCCAATGCGTCCT |  |
| In-vemR-F1 | TCCCCCGGGATGAGCAAACTCACCGTGCT | 380-bp fragment *vemR* with 6×His tag |
| his-vemR-R1 | TCAGTGGTGGTGGTGGTGGTGTTCCTGGCTCCTTACCGTC |  |
| his-vemR-F1 | CACCACCACCACCACCACTGAGTGAGTCCCGCATTCTGTT | 388-bp *vemR* with 6×His tag |
| In-vemR-R2 | GCTCTAGAGCCTGATGCTCGGCATCCAGCCGCT |  |
| HA-vemR-F | TCAAGCGTAATCTGGAACATCGTATGGGTATTCCTGGCTCCTTAC | 381-bp *vemR* with HA tag |
| HA-vemR-R | TACCCATACGATGTTCCAGATTACGCTTGAGTGAGTCCCGCATT |  |
| TAP-vemR-F | AAGCTTATGAGCAAACTCACCGTGC | 411-bp *vemR* with HA and Flag tags |
| TAP-vemR-R | GGTACCTCACTTGTCGTCGTCGTCCTTGTAGTCGACCTTGAGAGC |  |
| Flag-atoC-F | CCCAAGCTTATGAGTGAGTCCCGCATTCTGTTGA | 1509-bp *atoC* with Flag tag |
| Flag-atoC-R | GCGTCGACTCACTTGTCGTCGTCGTCCTTGTAGTCGTTGGCCAGCTCGGTCTG |  |
| Flag-cheA-F | GCGTCGACATGGACATGAACCAGCTGAT | 2022-bp *cheA* with Flag tag |
| Flag-cheA-R | GGGGTACCTCACTTGTCGTCGTCGTCCTTGTAGTCGGCCGCCAATGCGTCCTG |  |
| Flag-hrpG-F | CCC TTGACGCAGGACGCCCGCCTG | 750-bp *hrpG* with Flag tag |
| Flag-hrpG-R | GCGTCGACTCACTTGTCGTCGTCGTCCTTGTAGTCGGCAGGCGGCTGTGCGATGTGC |  |
| gyrB-F | CGGCACTTACGACTCCAGCAAG | 180-bp *gyrB* fragment for qPCR |
| gyrB-R | CCACCAGGATTTTCACCACGATG |  |
| hpa2-F | AGCAGAATCGAATGAATGGCATGCG | 145-bp *hpa2* fragment for qPCR |
| hpa2-R | CGTGCGTCAGTGTGTATGTAGCAGC |  |
| hrcC-F | TGGCAATCGCCGAAACGGTATCCAC | 200-bp *hrcC* fragment for qPCR |
| hrcC-R | CATGCTCAGGATCTGGGGTGCAA |  |
| hrcT-F | CTACGTCCTGAGCTCGTTCATCG | 210-bp *hrcT* fragment for qPCR |
| hrcT-R | GGGTTGGTCATCTGCACATTGTTG |  |
| hrpB7-F | ACAGCTCTACCTGCAGGGTCCAGCC | 121-bp *hrpB7* fragment for qPCR |
| hrpB7-R | GAAGGTGCCCTGGACGAGGCCAAAG |  |
| hrcN-F | GCATCGCTTCCCTGACGGTATTCGC | 171-bp *hrcN* fragment for qPCR |
| hrcN-R | CAAGAATCAGTACCCGGCAATCGAC |  |
| hrpB5-F | CGGATTCGTTCCACTCATCAAGCTG | 140-bp *hrpB5* fragment for qPCR |
| hrpB5-R | ACGTGCACAGACGCTGATCGACGAG |  |
| hrpB4-F | AGAACAGCGCACGCGCGATCAGGAC | 131-bp *hrpB4* fragment for qPCR |
| hrpB4-R | TATGCGGGTAATCCAGGCGTGGTCG |  |
| hrcJ-F | GCCAAACACAACGTCACCACACACC | 129-bp *hrcJ* fragment for qPCR |
| hrcJ-R | CTACGAAAACGTCAGTGTCACGCTG |  |
| hrpB2-F | CACGCCGACACTGACATTGAGGTTG | 97-bp *hrpB2* fragment for qPCR |
| hrpB2-R | ACCGACCATGGGGCTGCAGGAAATG |  |
| hrpB1-F | TGCTGACGAHCAACCGGTACGTCC | 172-bp *hrpB1* fragment for qPCR |
| hrpB1-R | GGCTGATCGAAGAAGACGCCGATGC |  |
| hrcU-F | GAGACACCGCTTGATATTGCC | 180-bp *hrcU* fragment for qPCR |
| hrcU-R | GCCCTCGCTTTCCTTGTATTC |  |
| hrcV-F | ACTGCCAACGCCCATGATCGATACG | 146-bp *hrcV* fragment for qPCR |
| hrcV-R | ACAACCGCAGTAGGGTGGTGAACAG |  |
| hpaP-F | CATCCTGCCAGTTGACGGCGCATTG | 175-bp *hpaP* fragment for qPCR |
| hpaP-R | ATCAGGTTCCGGTCGATCCTCGTCG |  |
| hrcQ-F | CTTACCCAATGATGCGGCTCGCCTG | 123-bp *hrcQ* fragment for qPCR |
| hrcQ-R | GAAACGCGTAGTGCCTCGTCGTCC |  |
| hrcR-F | AAGCACACACGGGAGCGCGAAAAGG | 112-bp *hrcR* fragment for qPCR |
| hrcR-R | CCGGTGCCAAGATAAGCAAGTCGTC |  |
| hrcS-F | AGCCTTGCTGCTCTGTCTGAAGGTG | 129-bp *hrcS* fragment for qPCR |
| hrcS-R | AGCTTGAGCGCGAACGAGATCGATG |  |
| hpaA-R | TGTCGTCGGTATGCGTGTFGCGGTC | 160-bp *hpaA* fragment for qPCR |
| hpaA-F | CAGGAGGATGAATTCGACGCCAACG |  |
| hrpD6-F | TGAGCAAGAȚTCTACAGACCAAGGC | 92-bp *hrpD6* fragment for qPCR |
| hrpD6-R | AATCTGTTGCGCAGTGGCATCGAGC |  |
| hrpD5-F | GATCCCCAAACCGATATACGAATAG | 223-bp *hrpD5* fragment for qPCR |
| hrpD5-R | ACTGGCTGAAACGCAACGAAGGATG |  |
| hrpE-F | CTTCGAACAGGGTATGGATGG | 162-bp *hrpE* fragment for qPCR |
| hrpE-R | TTGAGCTGCCTGATCHGTTG |  |
| hpaB-F | GGTAGAGGGCTTTGAGGTCT | 93-bp *hpaB* fragment for qPCR |
| hpaB-R | CCGTAAGCGATGCGTAAATACAGTG |  |
| hrpE3-F | CCAATATACGTTTGGTGTGAGATC | 80-bp *hrpE3* fragment for qPCR |
| hrpE3-R | CTGTCTGTGTTCTGTGCAGTGTCG |  |
| hpa4-F | CTGGCIȚGAGCAACAAGGTATTGAG | 187-bp *hpa4* fragment for qPCR |
| hpa4-R | GAGCCAGATCCACCACCGGTCATTC |  |
| hrpF-F | CAACGAGTCGTCTFCATCGGATCTG | 292-bp *hrpF* fragment for qPCR |
| hrpF-R | CGCATCCTTCCCCTTGATGTTCFTG |  |
| hpaF-F | CCAAGCAGGGTGTATCTCCATGTT | 184-bp *hpaF* fragment for qPCR |
| hpaF-R | CGTGTCTGCCGGTGATCTGCTTTCG |  |
| hrpG-F | GTTGCTCCGCGACGAAAATAC | 199-bp *hrpG* fragment for qPCR |
| hrpG-R | CTTGCGCAGCTTGTAGATATG |  |
| hrpX-F | GGCGAHGTTGTCTTHGCTC | 209-bp *hrpX* fragment for qPCR |
| hrpX-R | GACCTCATCGTCGGCTCCATC |  |
|  |  |  |
|  |  |  |
